# Elevated cortisol/DHEA ratio mediates the association between symptom severity and working memory impairment in drug-naive, first-episode OCD

**DOI:** 10.3389/fpsyt.2026.1771513

**Published:** 2026-02-24

**Authors:** Fangqing Song, Yuhan Li, Shaoxia Wang, Yanrong Wang, Jianqun Fang

**Affiliations:** 1 School of First Clinical, Ningxia Medical University, Yinchuan, Ningxia, China; 2 Institute of Medical Sciences, General Hospital of Ningxia Medical University, Yinchuan, Ningxia, China; 3 Mental Health Center, General Hospital of Ningxia Medical University, Yinchuan, Ningxia, China

**Keywords:** cortisol/DHEA ratio, executive function, mediation analysis, obsessive-compulsive disorder, response inhibition, working memory

## Abstract

**Objective:**

Dysregulation of the hypothalamic-pituitary-adrenal (HPA) axis has been implicated in the pathophysiology of obsessive-compulsive disorder (OCD) and its associated cognitive deficits. However, the role of the cortisol/dehydroepiandrosterone (DHEA) ratio, a functional index of the catabolic/anabolic balance, remains poorly characterized in the early stages of the illness. This study aimed to investigate the association between this neuroendocrine marker and executive function in a rigorously defined cohort of drug-naive, first-episode OCD patients.

**Methods:**

Seventy-five drug-naive, first-episode patients with OCD and 50 age- and sex-matched healthy controls (HCs) were recruited. Plasma concentrations of cortisol and DHEA were quantified by ELISA. Executive functions were assessed using a comprehensive battery, including response inhibition (Stop-Signal Task, SST), working memory (2-back task), and cognitive flexibility (Wisconsin Card Sorting Test, WCST). Data were analyzed using group comparisons, partial correlations, and stepwise multiple regression with Benjamini-Hochberg False Discovery Rate (FDR) correction. Mediation modeling was employed to explore mechanistic pathways.

**Results:**

Compared to HCs, the OCD group exhibited significant deficits in response inhibition and working memory, accompanied by elevated cortisol (*P* = 0.006), reduced DHEA (*P* < 0.001), and a markedly higher cortisol/DHEA ratio (*P* < 0.001). The elevated ratio was robustly correlated with greater symptom severity (Y-BOCS) and poorer inhibitory control (longer SSRT) after FDR correction (*P_adj_* < 0.05). Multiple regression analysis identified the cortisol/DHEA ratio as a significant independent predictor of response inhibition deficits (*P_adj_* = 0.002), whereas DHEA levels specifically predicted psychomotor slowing in working memory (*P_adj_* < 0.001). Critically, mediation analysis revealed that the cortisol/DHEA ratio showed a significant indirect effect on the relationship between symptom severity and working memory accuracy (95% *CI*: -0.0692 to -0.0096), suggesting a “suppression” effect where neuroendocrine dysregulation serves as a distinct biological pathway for cognitive impairment. No significant hormonal associations were observed for cognitive flexibility (*P* > 0.05).

**Conclusion:**

This study provides novel evidence that an elevated cortisol/DHEA ratio is not only a hallmark of drug-naive OCD but also independently predicts inhibitory dysfunction. Furthermore, this ratio appears to mediate the impact of clinical symptomatology on working memory capacity. These findings highlight the potential utility of the cortisol/DHEA ratio as a state-dependent biomarker and suggest that restoring the neuroendocrine balance could offer a therapeutic avenue for ameliorating cognitive deficits in OCD.

## 1 Introduction

Obsessive-compulsive disorder (OCD) is a debilitating psychiatric condition characterized by recurrent intrusive thoughts (obsessions) and repetitive ritualistic behaviors (compulsions) (1). Despite the established efficacy of first-line interventions, including selective serotonin reuptake inhibitors (SSRIs) and cognitive-behavioral therapy (CBT), a substantial proportion of patients exhibit partial or inadequate response, resulting in persistent functional impairment and a markedly diminished quality of life (2, 3). This therapeutic gap underscores the urgency of elucidating the neurobiological underpinnings of OCD to identify novel therapeutic targets (4, 5).

The pathophysiology of OCD is closely linked to dysregulation of the cortico-striatal-thalamic-cortical (CSTC) circuits, which are significantly modulated by the hypothalamic-pituitary-adrenal (HPA) axis (6). Although cortisol, the primary effector of the HPA axis, has been extensively studied (7–9), the contribution of other adrenal steroids remains largely underappreciated. Of particular interest is dehydroepiandrosterone (DHEA), a pivotal neurosteroid with well-documented mood-regulating properties (10, 11). While clinical studies of DHEA have primarily focused on other psychiatric conditions (12–14), preclinical evidence indicates that it enhances serotonergic neurotransmission in the striatum and amygdala—key structures within the dysregulated CSTC circuitry in OCD (15, 16).

Physiologically, cortisol and DHEA often exert functionally antagonistic effects (17). Therefore, their ratio may provide a more sensitive index of net glucocorticoid activity and HPA axis dysfunction than absolute levels of either hormone alone (18, 19). Notably, an altered cortisol/DHEA ratio has demonstrated superior discriminatory power in distinguishing individuals with depression from healthy controls, surpassing the diagnostic utility of absolute hormone levels (20, 21). Furthermore, clinical improvements have been correlated with a favorable shift in this ratio, independent of changes in cortisol alone (22). These findings suggest that the dynamic interplay between these hormones reflects unique biological information relevant to psychopathology.

In light of this, the present study investigates the association between the cortisol/DHEA ratio and both clinical symptomatology and key domains of executive function in a well-defined cohort of drug-naive patients with OCD. By eliminating the confounding effects of psychotropic medication, this study aims to: (1) evaluate the cortisol/DHEA ratio as a potential peripheral biomarker associated with cognitive deficits in OCD; (2) deepen the understanding of the neuroendocrine mechanisms underlying its pathophysiology; (3) provide a biological rationale for developing novel therapeutic strategies.

## 2 Materials and methods

### 2.1 Participant recruitment

Patients with drug-naive OCD were consecutively recruited from the outpatient psychiatry department of the General Hospital of Ningxia Medical University between January 2023 and June 2024. The sample size was determined *a priori* using power analysis. Based on previous literature, we anticipated a significant difference in cortisol/DHEA ratio between groups. Sample size was calculated a priori. Assuming an effect size derived from prior literature, with α = 0.05 and 1 - β = 0.80, a minimum of 45 participants per group was required. To account for potential participant dropout, we ultimately enrolled 75 patients with OCD and 50 healthy controls (HCs). The inclusion criteria for the OCD group were as follows: (1) age between 18 and 50 years; (2) meeting the Diagnostic and Statistical Manual of Mental Disorders, Fifth Edition (DSM-5) criteria for OCD, as confirmed by a senior psychiatrist; (3) a total Yale-Brown Obsessive Compulsive Scale (Y-BOCS) score ≥ 16; (4) no lifetime history of systematic pharmacotherapy or psychotherapy for OCD; (5) no first-degree family history of major psychiatric disorders; (6) right-handedness; (7) normal intellectual function, as estimated by clinical assessment. The HC group was matched to the patient group based on age, gender, and education level. The inclusion criteria for the HC group were identical to those of the patient group, with the exception of the OCD diagnosis. The exclusion criteria for all participants included the following: (1) a history of severe physical illnesses, endocrine disorders, immune-related diseases, or suicide attempts; (2) comorbid psychiatric disorders, including schizophrenia, bipolar disorder, intellectual disability, or alcohol dependence; (3) pregnancy or lactation. This study received approval from the Medical Research Ethics Committee of the General Hospital of Ningxia Medical University (approval number: KYLL-2023-0127). Written informed consent was obtained from all participants or their legal guardians.

### 2.2 Clinical diagnosis and assessment

Two psychiatrists who were trained to ensure inter-rater reliability conducted the clinical assessments using the Y-BOCS, the 17-item Hamilton Depression Rating Scale (HAMD-17), and the Hamilton Anxiety Rating Scale (HAMA). The HAMD-17 assesses depressive severity across seven factors, including anxiety/somatization and sleep disturbances. The HAMA is used to rate the severity of both somatic and psychological anxiety symptoms on a scale from 0 to 4. The Y-BOCS was employed to rate OCD severity based on the intensity of obsessive thoughts and compulsive behaviors, each item scored from 0 to 4. The Chinese version demonstrated good validity, with a Cronbach’s α of 0.83.

### 2.3 Cortisol and DHEA measurements

Peripheral venous blood samples were collected between 08:00 and 09:00 AM to control for circadian fluctuation. Participants were required to fast overnight (≥ 10 hours) and were instructed to abstain from strenuous exercise, caffeine, smoking, and alcohol for 24 hours prior to the procedure. Participants reporting acute sleep deprivation were rescheduled. Ethylenediaminetetraacetic acid (EDTA) was used as the anticoagulant. All steps were performed while maintaining the samples at 2 to 8°C. Samples were centrifuged at 1000 × g for 15 minutes within 30 minutes of collection. The resulting plasma supernatants were aliquoted and stored at -80°C until analysis to prevent repeated freeze-thaw cycles. Plasma concentrations of cortisol and DHEA were quantified using commercial Competitive Inhibition Enzyme-Linked Immunosorbent Assay (ELISA) kits (Jianglai Biology, Shanghai, China). For Cortisol (Catalog No. JL12377), the detection range was 0.312–20 ng/mL. For DHEA (Catalog No. JL12121), the detection range was 0.156–10 ng/mL. For both assays, the sensitivity was < 0.1 ng/mL. The intra-assay and inter-assay coefficients of variation were consistently < 9% and < 11%, respectively.

### 2.4 Executive function assessment

Response Inhibition (Stop-Signal Task, SST): Response inhibition was evaluated using a computerized Stop-Signal Task consisting of 240 trials divided into four blocks. The task employed a standard breakdown of 75% “Go” trials and 25% “Stop” trials. On Go trials, participants were instructed to respond as quickly and accurately as possible to a white arrow stimulus (1000 ms duration) by pressing “F” (left) or “J” (right). On Stop trials, a red arrow (the stop signal) appeared after a variable Stop-Signal Delay (SSD), cueing participants to withhold their response. The SSD was controlled by a dynamic staircase tracking algorithm targeting a 50% inhibition success rate: the SSD started at 250 ms and increased or decreased by 50 ms following a successful or failed inhibition, respectively. The primary outcome measure, Stop-Signal Reaction Time (SSRT), was calculated using the Integration Method to account for variations in inhibition probability. The probability of successful inhibition (Accuracy_stop) was also recorded.

Working Memory (2-Back Task): Working memory was assessed using a numerical 2-back task comprising two blocks of 60 trials each. Participants were presented with a sequence of digits (1–9) and required to indicate whether the current digit matched the one presented two steps earlier (approximate target frequency: 33%). To accommodate the psychomotor slowing and obsessive hesitation characteristic of unmedicated OCD patients, an extended temporal window was employed. Each trial consisted of a 500 ms stimulus presentation followed by a 4500 ms fixation interval (Total Stimulus Onset Asynchrony, SOA = 5000 ms). This design ensured the capture of valid but delayed responses. Performance was quantified by Accuracy (percentage of correct hits and correct rejections) and Mean Reaction Time (RT) for correct trials.

Cognitive Flexibility (Wisconsin Card Sorting Test, WCST): The standard computerized 128-card version of the WCST was administered to assess set-shifting and abstract reasoning. Participants matched response cards to four reference cards based on implicit rules (color, shape, or number) that changed automatically after 10 consecutive correct matches. Participants received feedback (Right/Wrong) after each sort. Key outcome measures included the number of Perseverative Errors (Rpe), reflecting the inability to shift sets after a rule change, and the number of Total Error Responses (Re), reflecting overall executive proficiency.

## 3 Data analysis

All statistical analyses were performed using SPSS version 27.0. Descriptive statistics, including means (M) and standard deviations (SD) for continuous variables and frequencies (n) and percentages (%) for categorical variables, were used to summarize the clinical and demographic characteristics of the participants. Group comparisons on demographic and clinical variables were performed using independent samples *t*-tests for continuous data and chi-square (*χ²*) tests for categorical data. To identify independent predictors of executive function performance, multiple linear regression analyses were conducted. Given the exploratory nature of the study, a stepwise selection procedure was primarily employed to maximize model parsimony and manage potential multicollinearity among clinical covariates (e.g., HAMA and HAMD). To ensure the robustness of these findings, key associations were further verified using a hierarchical regression approach. All models included hormonal markers as primary predictors, adjusting for potential confounders including sex, age, education level, and clinical scores. To control for the increased risk of Type I error due to multiple testing, *P*-values for correlation and regression analyses were adjusted using the Benjamini-Hochberg False Discovery Rate (FDR) procedure. Statistical significance was defined as an FDR-adjusted *P*-value < 0.05. Mediation analyses were performed using Model 4 of the PROCESS macro for SPSS (version 27.0) with 5000 bootstrap samples; a mediation effect was considered statistically significant if the bias-corrected 95% *CI* did not include zero.

## 4 Results

### 4.1 Demographic characteristics

No significant differences were found in gender, age, or education level between the OCD and HC groups (all *P* > 0.05). The OCD group (N = 75) had mean scores on the HAMA (15.84 ± 4.50) and HAMD (15.37 ± 4.50), indicating mild symptom levels, which were significantly higher than the HC group (HAMA: 7.56 ± 5.02, *P* < 0.001; HAMD: 6.42 ± 4.51, *P* < 0.001). Detailed data are presented in **Table 1**.

**Table 1:** Comparison of demographic data and executive function performance between the OCD group and the control group.

Variable	OCD (N = 75)	HC (N = 50)	*t/x^2^*	*P*
Gender (Male / Female)	34 / 41	21 / 29	0.135	0.713
Age (Year)	29.19 ± 11.72	29.60 ± 9.39	-0.218	0.828
Education (Years)	12.97 ± 3.42	14.06 ± 3.69	-1.660	0.094
Y-BOCS	24.27 ± 5.10	4.02 ± 3.65	24.407	< 0.001**
HAMA	15.84 ± 4.50	7.56 ± 5.02	9.415	< 0.001**
HAMD	15.37 ± 4.50	6.42 ± 4.51	10.886	< 0.001**
Cortisol (ng/mL)	68.68 ± 33.00	53.64 ± 23.70	2.963	0.006**
DHEA (ng/mL)	3.59 ± 0.85	4.32 ± 1.52	-3.091	< 0.001**
Cortisol/DHEA	20.14 ± 10.74	13.49 ± 7.16	4.15	< 0.001**
SST				
SSRT (ms)	203.30 ± 21.94	179.65 ± 10.32	8.089	< 0.001**
Accuracy	0.45 ± 0.25	0.52 ± 0.07	-2.331	0.041*
2-Back				
RT (ms)	3083.78 ± 759.77	2799.15 ± 601.39	2.329	0.022*
CC	15.61 ± 1.97	17.38 ± 1.07	-6.467	< 0.001**
WCST				
Re	40.59 ± 18.71	18.04 ± 14.04	7.686	< 0.001**
Rpe	20.85 ± 13.89	16.76 ± 12.21	1.737	0.093

Data are presented as mean ± SD. **P < 0.01; *P < 0.05.

OCD, obsessive-compulsive disorder; HC, healthy controls; Y-BOCS, Yale-Brown Obsessive-Compulsive Scale; HAMA, Hamilton Anxiety Scale; HAMD, Hamilton Depression Scale; SST, Stop Signal Task; SSRT, Stop Signal Reaction Time; RT, reaction time on 2-back task; CC, correct count on 2-back task; WCST, Wisconsin Card Sorting Test; Re, number of error responses; Rpe, number of persistent errors.

### 4.2 Comparison between patients and controls on cortisol, DHEA, cortisol/DHEA ratio, and executive function performance

Compared with the healthy control group, patients with OCD showed significantly elevated cortisol levels (*P* = 0.006), lower DHEA levels (*P* < 0.001), and a higher cortisol/DHEA ratio (*P* < 0.001). In terms of executive function, the OCD group also performed significantly worse across all domains assessed, including response inhibition, working memory, and cognitive flexibility (**Table 1**).

### 4.3 Correlation analysis

**Figure 1:**
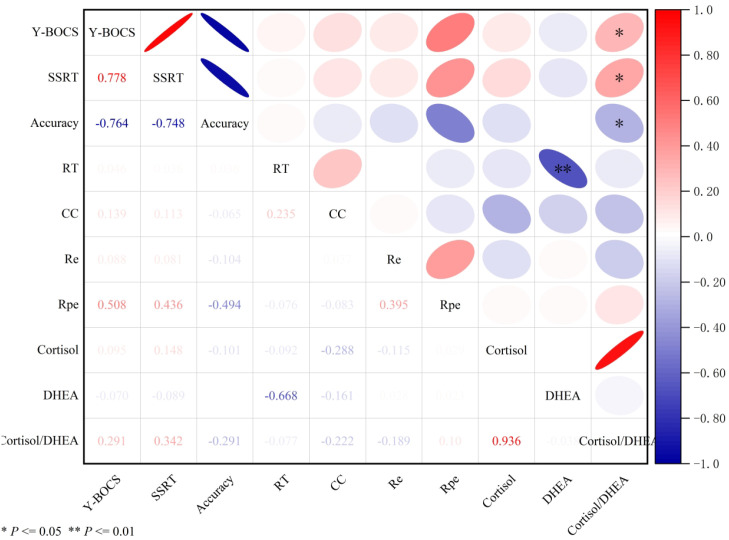
Pearson correlation matrix heatmap of hormonal biomarkers, OCD symptom severity, and cognitive performance. Pearson correlation matrix of hormonal biomarkers, OCD symptom severity, and cognitive performance measures. Analysis revealed that cortisol levels and the cortisol/DHEA ratio were positively associated with OCD symptom severity (Y-BOCS) and stop-signal reaction time (SSRT), indicating that a higher neuroendocrine stress load correlates with greater symptom burden and poorer inhibitory control. Conversely, these markers were inversely correlated with response inhibition accuracy (SST) and working memory accuracy (2-back). DHEA levels generally demonstrated an opposing, protective profile, correlating negatively with symptom severity and positively with cognitive accuracy. Consistent with the group comparisons, no significant associations were observed between hormonal measures and Wisconsin Card Sorting Test (WCST) performance. Values represent Pearson correlation coefficients (r). To control for Type I error inflation, all *P*-values were adjusted using the Benjamini-Hochberg False Discovery Rate (FDR) method. **P_adj_ < 0.01,*P_adj_ < 0.05. OCD, obsessive-compulsive disorder; HC, healthy controls; Y-BOCS, Yale-Brown Obsessive-Compulsive Scale; HAMA, Hamilton Anxiety Scale; HAMD, Hamilton Depression Scale; SST, Stop Signal Task; SSRT, Stop Signal Reaction Time; RT, reaction time on 2-back task; CC, correct count on 2-back task; WCST, Wisconsin Card Sorting Test; Re, number of error responses; Rpe, number of persistent errors.

To control for Type I errors due to multiple testing, *P*-values were adjusted using the Benjamini-Hochberg False Discovery Rate (FDR) method, stratified by a priori hypotheses (confirmatory vs. exploratory). In the confirmatory analysis, the cortisol/DHEA ratio remained significantly positively correlated with OCD symptom severity (Y-BOCS: r = 0.291, *P_adj_* = 0.019). Regarding executive function, the ratio demonstrated a robust association with response inhibition deficits, showing a positive correlation with SSRT (r = 0.342, *P_adj_* = 0.014) and a negative correlation with inhibition accuracy (r = -0.291, *P_adj_* = 0.019). For working memory (2-back task), the negative correlation between the ratio and accuracy approached significance after correction (r = -0.222, *P_adj_* = 0.070). In the exploratory analysis of individual hormones, DHEA levels exhibited a highly significant negative correlation with working memory reaction time (r = -0.668, *P_adj_* < 0.001), suggesting a specific role in psychomotor processing speed. Other bivariate correlations involving cortisol alone did not survive the stringent FDR correction (*P_adj_* > 0.05). Consistent with the unadjusted results, no significant associations were observed between any hormonal measures and performance on the WCST (all *P_adj_* > 0.05) (**Table 2**, **Figure 1**).

**Table 2:** The Correlation between the main variables.

Variable	1	2	3	4	5	6	7	8	9	10
1.Y-BOCS	1									
2.SSRT	0.778	1								
3.Accuracy	-0.764	-0.748	1							
4.RT	0.046	0.036	0.036	1						
5.CC	0.139	0.113	-0.065	0.235	1					
6.Re	0.088	0.081	-0.104	0.016	0.037	1				
7.Rpe	0.508	0.436	-0.494	-0.076	-0.083	0.395	1			
8.Cortisol	0.095	0.148	-0.101	-0.092	-0.288	-0.115	0.029	1		
9.DHEA	-0.070	-0.089	0.006	-0.668**	-0.161	0.028	0.023	0.011	1	
10.Cortisol/DHEA	0.291*	0.342*	-0.291*	-0.077	-0.222	-0.189	0.100	0.936	-0.038	1

**P_adj_ < 0.01; *P_adj_ < 0.05.

OCD, obsessive-compulsive disorder; HC, healthy controls; Y-BOCS, Yale-Brown Obsessive-Compulsive Scale; HAMA, Hamilton Anxiety Scale; HAMD, Hamilton Depression Scale; SST, Stop Signal Task; SSRT, Stop Signal Reaction Time; RT, reaction time on 2-back task; CC, correct count on 2-back task; WCST, Wisconsin Card Sorting Test; Re, number of error responses; Rpe, number of persistent errors.

### 4.4 Predictors of executive functioning in OCD

To identify independent predictors of executive functioning in OCD, we performed stepwise multiple linear regression analyses for each executive domain. Three separate models were constructed for each task, featuring one primary predictor (cortisol, DHEA, or the cortisol/DHEA ratio) while controlling for sex, age, education, and clinical scores (HAMA, HAMD). To ensure statistical rigor, *P*-values for hormonal predictors were adjusted using the False Discovery Rate (FDR) method.

For the SST, a higher education level was a significant predictor of better performance across models. Notably, the cortisol/DHEA ratio remained a significant independent predictor of inhibitory deficits even after controlling for covariates and applying FDR correction. Specifically, a higher ratio significantly predicted both poorer inhibition speed (longer SSRT, *P_adj_* = 0.002) and reduced inhibition accuracy (*P_adj_* = 0.004).

For the 2-back task, the regression results highlighted a domain-specific role of DHEA. Unlike the findings for response inhibition, the cortisol/DHEA ratio was not a significant predictor of working memory performance in the multivariate models (*P_adj_* > 0.05). Instead, DHEA levels emerged as the strongest and most robust predictor of working memory processing speed. Lower DHEA levels significantly predicted longer reaction times (*P_adj_* < 0.001), suggesting that DHEA deficits may specifically underlie psychomotor slowing in OCD. The association between cortisol and working memory accuracy, while observed in unadjusted analyses, did not survive FDR correction (*P_adj_* > 0.05).

Analysis of the WCST focused on Re, as Rpe did not differ between groups. Higher education was a significant predictor of fewer errors across all models (*P* < 0.05). However, consistent with the correlation results, none of the hormonal measures (cortisol, DHEA, or the ratio) were significant predictors of cognitive flexibility performance (**Table 3**).

**Table 3:** Stepwise multiple regression analyses for executive functioning, including clinicodemographic variables as well as cortisol, DHEA, or cortisol/DHEA ratio as predictors.

DependentVariable	Predictors	Standardized β	*t*-value	*P*-value
SST - SSRT (ms)	Cortisol/DHEA	0.426	3.775	0.002**
SST - Accuracy	Cortisol/DHEA	-0.375	-3.335	0.004**
2-Back - RT (ms)	DHEA	-0.632	-7.535	< 0.001**
2-Back - CC (Count)	Cortisol	-0.222	-2.030	0.070
WCST - Re (Errors)	(All *P* > 0.05)			

Data are presented as mean. **P_adj_ < 0.01.

SST, Stop Signal Task; SSRT, Stop Signal Reaction Time; RT, reaction time on 2-back task; CC, correct count on 2-back task; WCST, Wisconsin Card Sorting Test; Re, number of error responses.

### 4.5 Mediation analysis of the cortisol/DHEA ratio on OCD symptoms and cognitive functions

**Figure 2:**
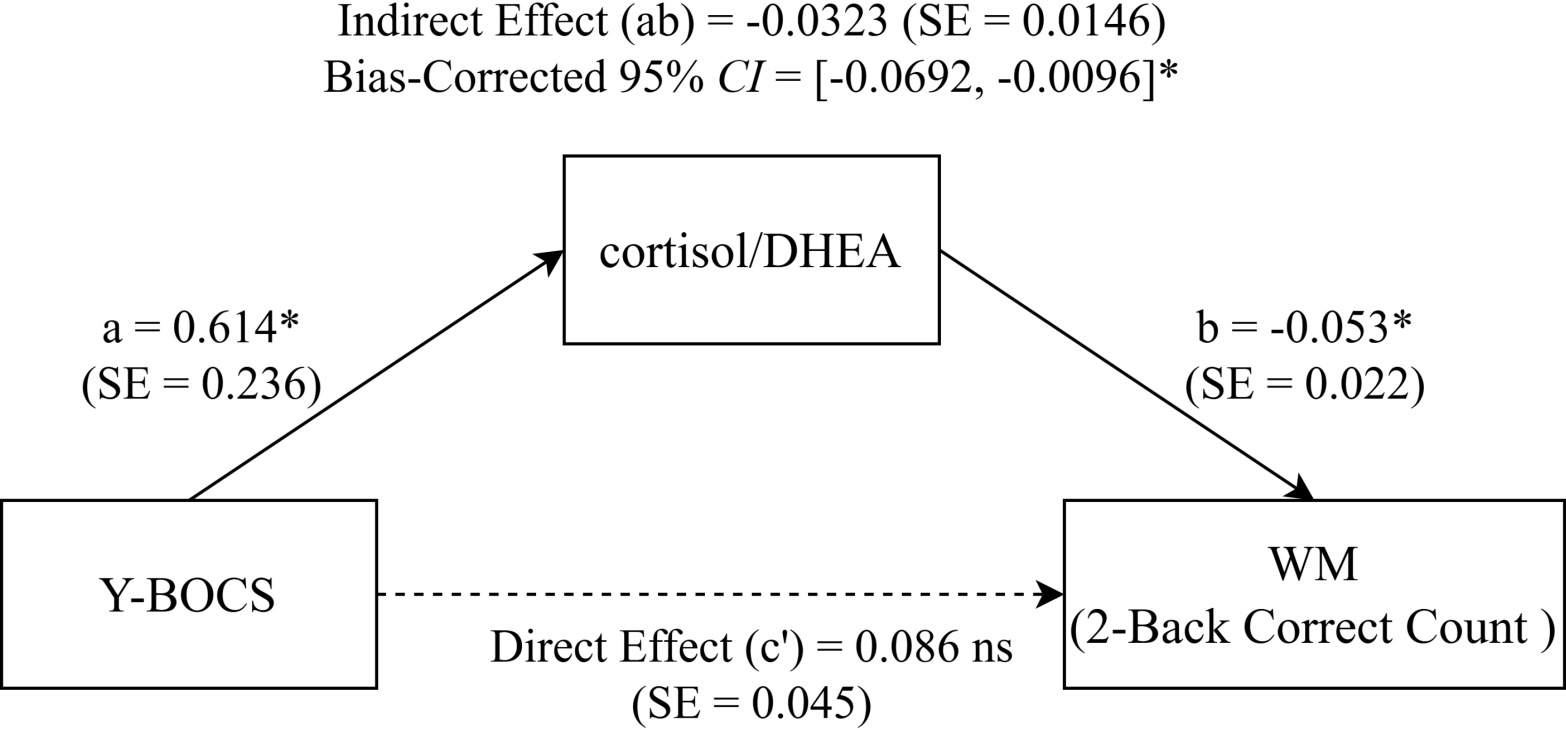
Path Coefficients for the Cortisol/DHEA Ratio in the Association Between OCD Severity and Working Memory Performance. Statistical Mediation Model. Path diagram illustrating the mediating role of the Cortisol/DHEA ratio in the relationship between OCD symptom severity (Y-BOCS) and Working Memory (2-Back Correct Count). Values on paths represent unstandardized regression coefficients **(B)** with standard errors (SE) in parentheses. The solid lines indicate significant pathways (*P < 0.05), while the dashed line indicates a non-significant direct effect (c’). The box at the top reports the significant indirect effect (a × b) with its bias-corrected 95% bootstrap confidence interval. *P < 0.05. Y-BOCS, Yale-Brown Obsessive-Compulsive Scale; WM, Working Memory; Cortisol/DHEA, cortisol-to-dehydroepiandrosterone ratio.

The mediation analysis indicated no significant mediating role of the cortisol/DHEA ratio in the relationship between OCD symptoms and response inhibition, since the 95% *CI* for the indirect effect included zero. Conversely, a significant indirect effect of the cortisol/DHEA ratio on the association between OCD symptoms and working memory accuracy was observed (bias-corrected 95% *CI* = -0.0692 to -0.0096, excluding zero). Decomposition of effects showed that while the direct effect (Effect = 0.0862) and total effect (Effect = 0.0539) were not statistically significant, the indirect effect via the ratio remained significant (Effect = -0.0323; SE = 0.0146). This pattern of results (**Table 4**, **Figure 2**) suggests that OCD symptoms impair working memory specifically through the indirect pathway of HPA axis dysregulation, consistent with a suppression model where the specific neuroendocrine impact is masked in the total association.

**Table 4:** Path coefficients for the cortisol/DHEA ratio in the association between OCD severity and working memory performance.

Path	Y-BOCS – Cortisol/DHEA – WM			
Effect	Effect value	SE	LLCI	ULCI
Total effect	0.054	0.045	-0.035	0.143
Direct effect	0.086	0.045	-0.004	0.177
Indirect effect	-0.032	0.015	-0.069	-0.010

Y-BOCS, Yale-Brown Obsessive-Compulsive Scale; WM, Working Memory; Cortisol/DHEA, the cortisol to dehydroepiandrosterone ratio.

## 5 Discussion

To the best of our knowledge, this is the first study to comprehensively evaluate DHEA levels, the dynamics of the peripheral cortisol/DHEA ratio, and their collective association with specific domains of executive function in a well-defined cohort of drug-naive, first-episode OCD patients. This research provides a preliminary empirical framework for elucidating the neuroendocrine underpinnings of OCD, utilizing peripheral biomarkers to probe central pathophysiology.

Our primary findings corroborate dysregulated cortisol levels in OCD patients (8, 9), and their normalization often correlates with successful therapeutic outcomes (23). Consistent with this, we observed significantly lower DHEA levels in our cohort of first-episode, drug-naive patients. A central thesis of this study is that the cortisol/DHEA ratio may offer a more functionally relevant index of HPA axis activity than either hormone in isolation. This ratio is conceptualized as an indicator of the “anabolic/catabolic balance,” reflecting the net antagonism between the neuroprotective properties of DHEA and the neurotoxic actions of cortisol (24, 25). Supporting this thesis, our analysis revealed a significant positive correlation between the cortisol/DHEA ratio and OCD symptom severity. This aligns with existing evidence linking an elevated ratio to an increased risk for stress-related psychopathologies (26, 27).

Regarding the association with cognition, the robust correlation observed between inhibitory control deficits (SSRT) and symptom severity (r = 0.778) appears to be specific to the OCD cohort. In our analysis, the healthy control group showed no significant association between these measures. This dissociation suggests that the strong coupling between cognitive slowing and symptom burden is a pathological feature of the disorder’s acute, unmedicated state (‘generalized inhibitory collapse’), rather than a generalized physiological trait inherent to the cognitive task itself. Notably, the cortisol/DHEA ratio emerged as a significant independent predictor of response inhibition deficits in our multiple regression model. This finding provides novel clinical evidence from an OCD cohort that reinforces established neurobiological models of stress-induced prefrontal cortex dysfunction (28, 29).

Furthermore, our mediation analysis revealed a significant indirect effect, indicating that the cortisol/DHEA ratio mediates the relationship between OCD symptom severity and working memory performance. Interestingly, while the total effect of symptom severity on working memory was not statistically significant, the indirect effect via the cortisol/DHEA ratio was robust and negative. This pattern, statistically referred to as “inconsistent mediation” (30), suggests that the cognitive impact of OCD is nuanced. Specifically, this finding implies that OCD symptoms per se (e.g., behavioral rituals) may not linearly predict working memory deficits in surface-level analyses, possibly due to compensatory cognitive control or the increased mental effort often exerted by high-functioning patients to maintain performance (31). However, the physiological cost associated with this chronic distress—manifesting as HPA axis dysregulation (elevated cortisol/DHEA ratio)—exerts a specific and significant deleterious effect on working memory capacity. Thus, the HPA axis imbalance acts as a distinct “biological brake” on cognition. We also rigorously assessed the potential confounding influence of speed-accuracy trade-offs (SAT). Our data argue against a hypothesis of strategic slowing; instead, the OCD group exhibited a ‘double deficit’ profile, characterized by both significantly prolonged reaction times and reduced accuracy compared to healthy controls (**Table 1**). Furthermore, the absence of a significant correlation between reaction time and accuracy within the OCD cohort following FDR correction (*P_adj_* > 0.05) suggests that psychomotor slowing and executive capacity limitations represent distinct, parallel impairments rather than a compensatory functional trade-off.

These results align with and extend previous research linking neuroactive steroid ratios to cognitive function in other psychiatric disorders. For instance, Silver et al. (32) demonstrated that DHEA levels correlate with cognitive performance in chronic schizophrenia, while Jin et al. (33) linked elevated cortisol/DHEA ratios to reduced hippocampal volume in major depression.

A key finding that warrants specific contextualization is the reduced DHEA level in our cohort. Approximately 80% of circulating DHEA is of adrenal origin, with the remainder synthesized in the gonads and brain (34). Unlike cortisol, DHEA exhibits markedly attenuated diurnal rhythmicity and lacks a significant cortisol awakening response (35), properties that may confer greater stability for biomarker assessment. We specifically chose to measure plasma DHEA rather than its sulfated form (DHEA-S), despite the latter’s higher stability. This decision was based on the lipophilic nature of DHEA, which allows it to readily cross the blood-brain barrier and exert direct neuroactive effects, making it a more immediate proxy for central activity during cognitive tasks compared to the hydrophilic DHEA-S.

Notably, our finding of reduced DHEA contrasts with reports from Erbay et al. describing elevated DHEA in OCD (36, 37). In this model, the cyclical nature of obsessions and compulsions perpetuates a state of persistent distress and low-grade HPA axis activation. The observed elevation in the cortisol/DHEA ratio may reflect a hypothesized functional shift in adrenal steroidogenesis. Under chronic stress, it has been postulated that precursor resources are preferentially diverted towards cortisol synthesis at the expense of DHEA, a phenomenon potentially involving alterations in CYP17A1 enzymatic activity (38). This would mechanistically account for the concurrent findings of elevated cortisol and reduced DHEA in our patient cohort.

While our cross-sectional design necessitates caution, these empirical associations align with established neurobiological models. The established neuroanatomical abnormalities in OCD, notably within the cortico-striatal-thalamic-cortical (CSTC) circuit, hippocampus, and amygdala (39), involve regions known to be particularly sensitive to hypercortisolemia. Anatomically, the prefrontal cortex and hippocampus possess a high density of glucocorticoid receptors (40–42), and cortisol readily crosses the blood-brain barrier (43) to mediate structural alterations (44).

We speculate that an elevated Ratio—reflecting net glucocorticoid activity—may conduce to a state of “neurotoxicity”. Previous preclinical literature indicates that at a cellular level, chronically elevated glucocorticoids can exert neurotoxic effects, such as reducing glial cell proliferation (45) and impairing dendritic morphology (46–48). Conversely, DHEA is known to be neurotrophic and neuroprotective, promoting neurogenesis and neurite outgrowth, while also exerting antioxidant effects (6, 49–53). Given DHEA’s established anti-glucocorticoid properties (54), a deficit in DHEA may conduce to a state of diminished neuroprotection and dysregulated neurotransmission (involving cholinergic, dopaminergic, and glutamatergic systems (55)).

**Figure 3:**
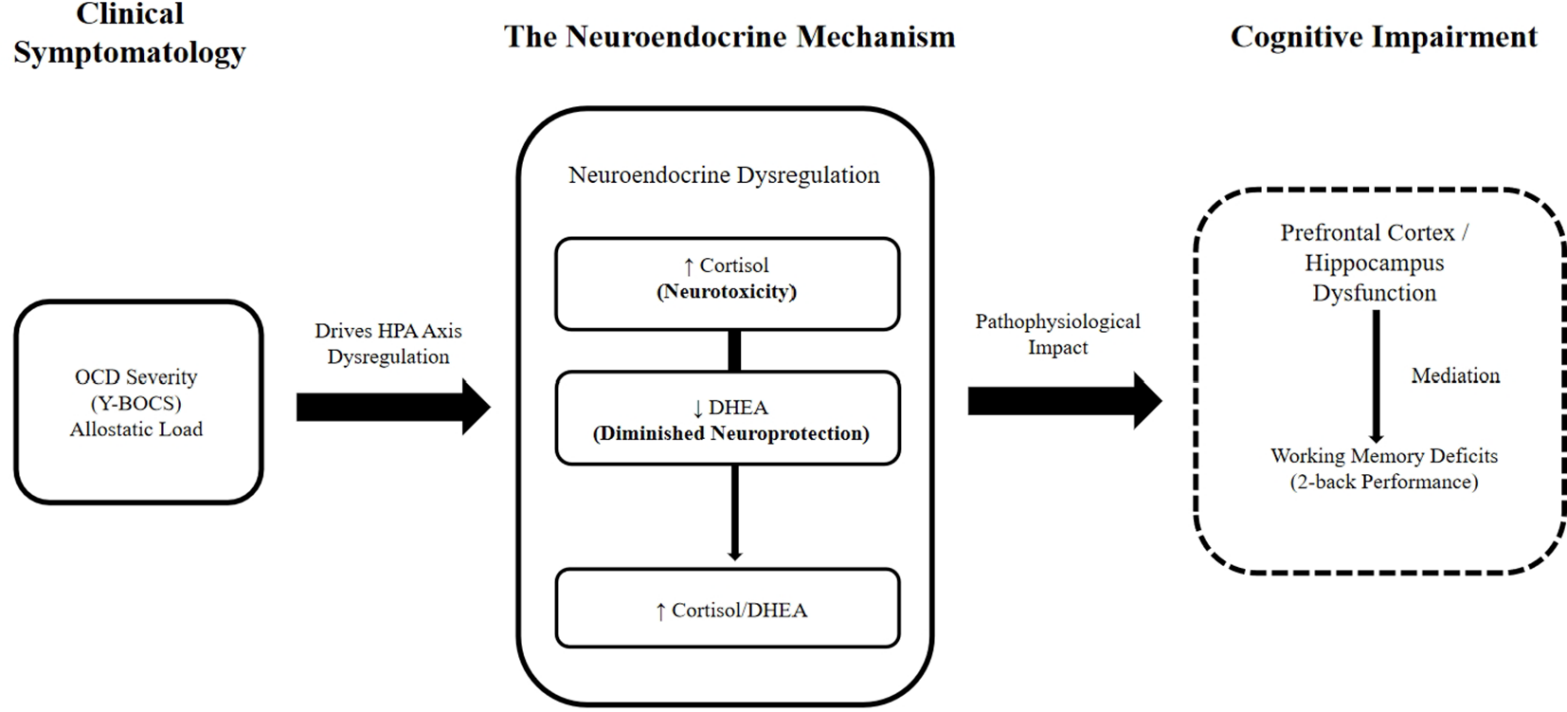
Hypothesized conceptual model illustrating the pathophysiological link between OCD symptomatology, HPA-axis dysregulation, and executive dysfunction. The schematic proposes that the chronic distress and allostatic load associated with OCD symptom severity drive a maladaptive shift in the HPA axis, resulting in an elevated Cortisol/DHEA ratio. This neuroendocrine imbalance—characterized by excessive glucocorticoid activity relative to neuroprotective DHEA—is hypothesized to exert neurotoxic effects on glucocorticoid-sensitive brain regions, particularly within frontostriatal circuits (e.g., Dorsolateral Prefrontal Cortex). The resulting compromise in neural integrity ultimately manifests as broad executive dysfunction, encompassing specific deficits in both working memory and response inhibition.

Based on these findings, we postulate that similar sustained neuroendocrine imbalances in OCD may impair the functional integrity of frontostriatal circuits, thereby mediating the observed deficits in response inhibition and working memory, consistent with prior findings in related disorders. While prior studies in healthy individuals and patients with schizophrenia have linked DHEA to improved cognitive functions like working memory and attention (12, 56, 57), our findings provide the first clinical evidence in an OCD cohort to support this model. Additionally, preclinical evidence indicates that DHEA modulates central neurotransmitter systems, increasing serotonin turnover (15) and upregulating 5-HT2A receptor expression (16). Importantly, the anatomical alignment between DHEA-modulated regions and core nodes of the CSTC circuit provides a compelling neurobiological rationale for investigating DHEA’s role in OCD (**Figure 3**).

Finally, regarding the null findings for cognitive flexibility, while the lack of significant correlations between hormonal ratios and WCST performance might imply a domain-specific vulnerability of inhibitory and working memory circuits, methodological nuances warrant consideration. Crucially, the executive profile of our first-episode, drug-naive cohort appears distinct from chronic OCD populations. The non-significant group difference in perseverative errors suggests that set-shifting deficits may be subtler or preserved in the early stages of the illness relative to the robust impairments observed in inhibition and working memory. Consequently, the absence of hormonal correlations with WCST performance could reflect limited psychometric sensitivity to these mild deficits or range restriction in error rates among high-functioning patients, rather than a definitive absence of biological association.

Several limitations of the present study warrant careful consideration when interpreting the results. First, the study’s cross-sectional design precludes any definitive causal inferences. While our mediation analysis provides statistical support for the involvement of the cortisol/DHEA ratio in working memory deficits, we cannot rule out the possibility of reverse causality, where cognitive deficits induce stress and hormonal dysregulation, or bidirectional relationships. Longitudinal designs are essential to clarify the temporal sequence of these associations. Second, although our sample size was sufficient for the main analyses, it was relatively modest. Third, we measured peripheral hormone levels in plasma, which may not fully capture central neurosteroid activity. Furthermore, while we standardized sampling to a narrow morning window (08:00 - 09:00 AM) to control for clock time, we did not account for the time elapsed since awakening (CAR). Moreover, elevated cortisol lacks specificity for OCD, as it has also been documented in other major psychiatric disorders. Fourth, potential confounders like BMI, objective sleep quality, physical activity levels, and a history of early life stress were not assessed. Fifth, subthreshold affective symptoms may still contribute to HPA variance. Additionally, while stepwise regression was used for model parsimony, we acknowledge its potential for overfitting and Type I error inflation, although our key findings were robust to hierarchical validation.

Finally, to ensure internal validity, we applied stringent exclusion criteria. Future longitudinal studies should examine the dynamic trajectory of the cortisol/DHEA ratio following first-line interventions (e.g., SSRIs or CBT). Determining whether ratio normalization tracks with symptom remission, or if baseline deviations predict treatment resistance, would validate its potential as a state-dependent biomarker and facilitate neuroendocrine-guided precision medicine in OCD.

## Data Availability

The raw data supporting the conclusions of this article will be made available by the authors, without undue reservation.
